# ADULT FOSTER HOME OWNERS' PERSPECTIVES ON REWARDS AND CHALLENGES OF OPERATING AN ADULT FOSTER HOME

**DOI:** 10.1093/geroni/igz038.3499

**Published:** 2019-11-08

**Authors:** Sheryl Elliott, Sarah Dys, Jaclyn Winfree, Paula Carder

**Affiliations:** 1 The Institute on Aging at Portland State University, Portland, Oregon, United States; 2 Institute on Aging, Portland, Oregon, United States; 3 Portland State University, Portland, Oregon, United States; 4 Portland State University Institute on Aging, Portland, Oregon, United States

## Abstract

Adult foster homes (AFHs) are small, residential settings providing older adults and persons with disabilities an alternative to nursing facilities and larger residential care settings. Some groups, including individuals with Alzheimer’s disease and related dementias, are well served by smaller settings. Although AFHs are common throughout the US, research on this setting is scant and dated. This study summarizes four years of qualitative data from Oregon AFH owners’ (N=726) responses to open-ended questions about the challenges and rewards of owning and operating an AFH. Content analysis of 924 comments indicate that providing resident care (42%), finding the work meaningful and “a life calling” (21%), developing a family-like connection with residents (15%), and working at home (8%) were the most commonly reported rewards. The most frequently described challenges included caring for residents with multiple chronic health conditions—including those with difficult behaviors (17%), difficulty hiring and retaining qualified caregivers (15%), low Medicaid reimbursement rates (14%), and adhering to administrative rules (14%). Results highlight AFH providers’ personal satisfaction with caring for and establishing connections with residents, and challenges associated with residents’ increasingly complex care needs, Medicaid reimbursement rates, and attitudes about state regulations. Although AFHs are licensed by states, they are subject to federal regulations, including the 2014 Centers for Medicare & Medicaid Services home and community-based services ruling. The new regulations, Oregon administrative rules, Medicaid reimbursement rates, and caregiver supply are presented to contextualize AFH owner comments and regulatory considerations.

